# The frontline antibiotic vancomycin induces a zinc starvation response in bacteria by binding to Zn(II)

**DOI:** 10.1038/srep19602

**Published:** 2016-01-22

**Authors:** Ashraf Zarkan, Heather-Rose Macklyne, Andrew W. Truman, Andrew R. Hesketh, Hee-Jeon Hong

**Affiliations:** 1 Department of Biochemistry, University of Cambridge, Cambridge, CB2 1QW, UK; 2 Department of Molecular Microbiology, John Innes Centre, Norwich, NR4 7UH, UK; 3 Cambridge Systems Biology Centre, University of Cambridge, Cambridge, CB2 1QW, UK

## Abstract

Vancomycin is a front-line antibiotic used for the treatment of nosocomial infections, particularly those caused by methicillin-resistant *Staphylococcus aureus*. Despite its clinical importance the global effects of vancomycin exposure on bacterial physiology are poorly understood. In a previous transcriptomic analysis we identified a number of Zur regulon genes which were highly but transiently up-regulated by vancomycin in *Streptomyces coelicolor*. Here, we show that vancomycin also induces similar zinc homeostasis systems in a range of other bacteria and demonstrate that vancomycin binds to Zn(II) *in vitro*. This implies that vancomycin treatment sequesters zinc from bacterial cells thereby triggering a Zur-dependent zinc starvation response. The Kd value of the binding between vancomycin and Zn(II) was calculated using a novel fluorometric assay, and NMR was used to identify the binding site. These findings highlight a new biologically relevant aspect of the chemical property of vancomycin as a zinc chelator.

The glycopeptide antibiotic vancomycin was first discovered in the 1950’s^1^. Since that time it has been reserved as an antibiotic of last resort for the treatment of multiple drug-resistant Gram-positive infectious agents, particularly those resistant to β-lactam family antibiotics such as methicillin-resistant *Staphylococcus aureus*. However, in common with other antibiotics, resistance to vancomycin has emerged in bacterial populations^2^. In addition, its use has also had to be carefully managed owing to toxic side-effects^3^. The relationship between genotype and phenotype in vancomycin resistant bacteria, and into the global response of cultures exposed to vancomycin, has been the subject of extensive research^4^. The motivation behind these and similar studies is to obtain an integrated understanding of how bacteria can adapt to survive in the presence of antibiotic treatments and thereby to suggest opportunities for improving activity via rational redesign, or by synergistically targeting more than one important cellular process.

We have previously undertaken an extensive transcriptome profiling study analyzing the effect of sub-inhibitory concentrations of three antibiotics, vancomycin, bacitracin and moenomycin A, that target distinctly different stages of cell wall biosynthesis in *Streptomyces coelicolor*^5^. This model actinomycete is non-pathogenic and does not synthesize any glycopeptide antibiotic but possesses an inducible vancomycin resistance cluster that serves as a useful model for mechanistic investigations into high-level vancomycin resistance^6^,^7^,^8^,^9^. In addition to the expected up-regulation in expression of the seven *van* resistance cluster genes, a set of genes under the control of Zur were also found to be strongly up-regulated in response only to vancomycin (Fig. 1a)^10^. Zur is a regulatory protein which represses the expression of its target genes when bound to Zn(II) but permits transcription in its zinc-free form (Fig. 1b). It thus functions as an intracellular zinc responsive switch controlling the expression of genes involved in zinc homeostasis^11^,^12^. Zinc is an important catalytic or structural cofactor required for the activity of a wide variety of proteins but it is also toxic in excess and its intracellular concentration must be maintained within limits appropriate for viability. Zinc homeostasis plays a significant role in pathogenesis where bacteria need to successfully compete for limited Zn(II) within their host while also being able to survive antimicrobial concentrations of the metal that can be deliberately produced at sites of infection^13^.

The simplest explanation for the observation that vancomycin treatment causes a zinc depletion response in *S. ceolicolor* cells is that the antibiotic directly chelates Zn(II), analogous to its known interaction with Cu(II)^14^. This raises the possibility that zinc homeostasis is important for adaptation to antibiotic therapy in addition to host colonization. Here we investigate this hypothesis and report that vancomycin indeed acts as a weak zinc chelator that is capable of inducing the activity of Zur-dependent zinc homeostasis mechanisms in a range of bacteria. We determine the binding affinity of vancomycin for Zn(II) and define a potential binding site.

## Results

### Vancomycin induces Zur-dependent zinc responsive mechanisms in different bacterial species

**Figure 2:**
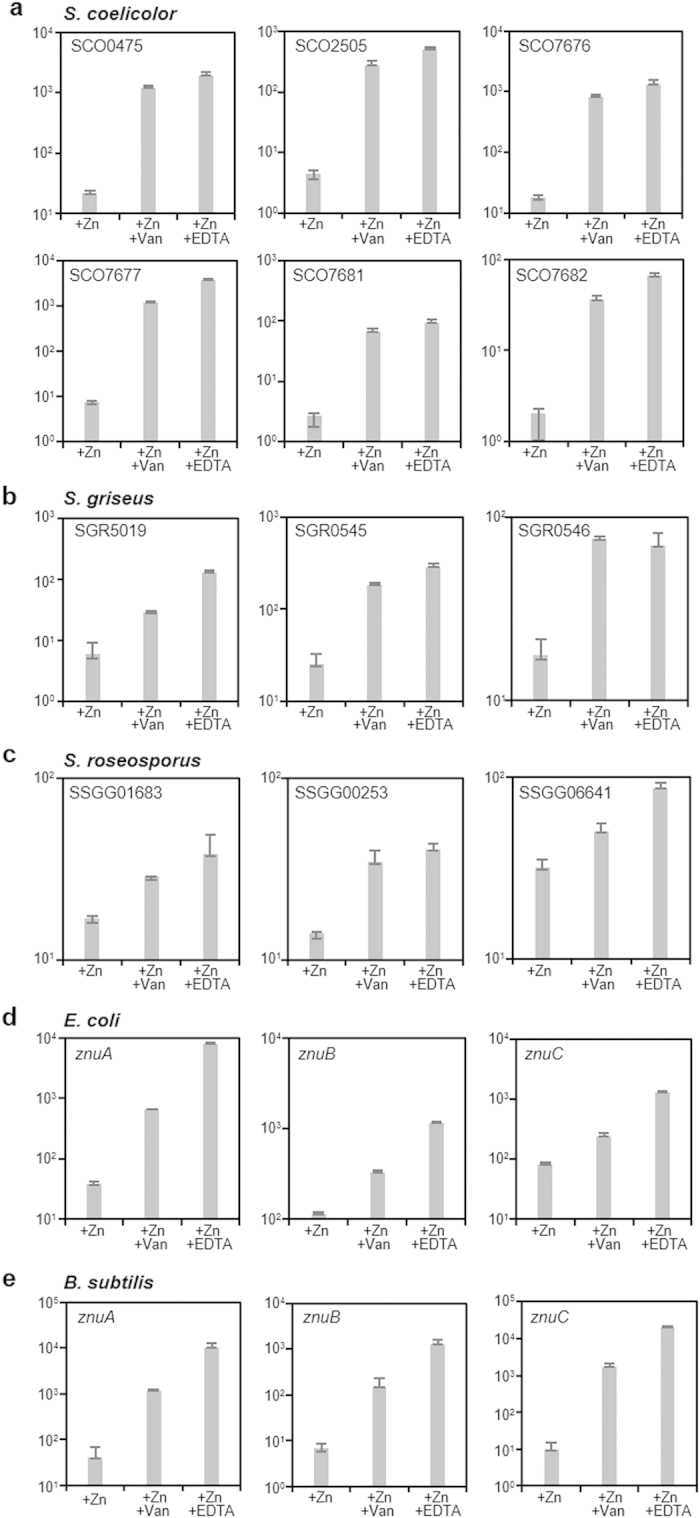
Vancomycin induces Zur-dependent zinc responsive mechanisms in different bacterial species. qRT-PCR analysis of the transcriptional response to vancomycin of (**a**) the six Zur-regulated genes SCO0475, SCO2505, SCO7676, SCO7677, SCO7681 and SCO7682 in *S. coelicolor* (**b**) the putative Zur regulated genes SGR5019 (SCO2505 orthologue), SGR0545 (SCO3429 orthologue) and SGR0546 (SCO3428 orthologue) in *S. griseus* (**c**) the putative Zur regulated genes SSGG01683 (SCO2505 orthologue), SSGG00253 (SCO0476 orthologue) and SSGG06641 (SCO3428 orthologue) in *S. roseosporus* (**d**) *znuA*, *znuB* and *znuC* from the zinc transport system in *E. coli* and (**e**) *B. subtilis znuABC*. “+Zn” indicates a sample from a culture grown in the presence of ~3 μM of zinc sulphate, and “+Zn +Van” or “+Zn +EDTA” indicates a sample from similar cultures treated with 2 mM of either vancomycin or EDTA for 30 min. The *y* axis indicates transcript abundance normalized to a reference gene presented in a log scale. Data are presented as means ± SD.

To investigate the ability of vancomycin to induce a zinc starvation response in different species of bacteria, cultures of *S. coelicolor*, *Streptomyces griseus*, *Streptomyces roseosporus*, *Escherichia coli* and *Bacillus subtilis* were treated with vancomycin for 30 min and changes in transcription of Zur-regulated genes quantified using qRT-PCR (Fig. 2). The response to exposure to an equivalent dose of the metal ion chelator EDTA was used as a positive control. In *S. coelicolor*, six representative Zur-regulated genes showed a strong (ca. 10 to 100 fold) up-regulation in response to both EDTA and vancomycin treatment in comparison to the untreated control (Fig. 2a), confirming the observations from the previous transcriptome analysis^5^. Three putative Zur-regulated genes identified by bioinformatics analysis in two additional *Streptomyces* strains, *S. griseus* and *S. roseosporus,* also showed a similar pattern of induction indicative of zinc starvation in response to vancomycin in these species (Fig. 2b,c). Transcription of each of the *znuABC* genes in *E. coli*^15^ and *B. subtilis*^16^ was also markedly up-regulated in response to vancomycin treatment (Fig. 2d,e).

### Vancomycin binds to Zn(II) *in vitro*

**Figure 3:**
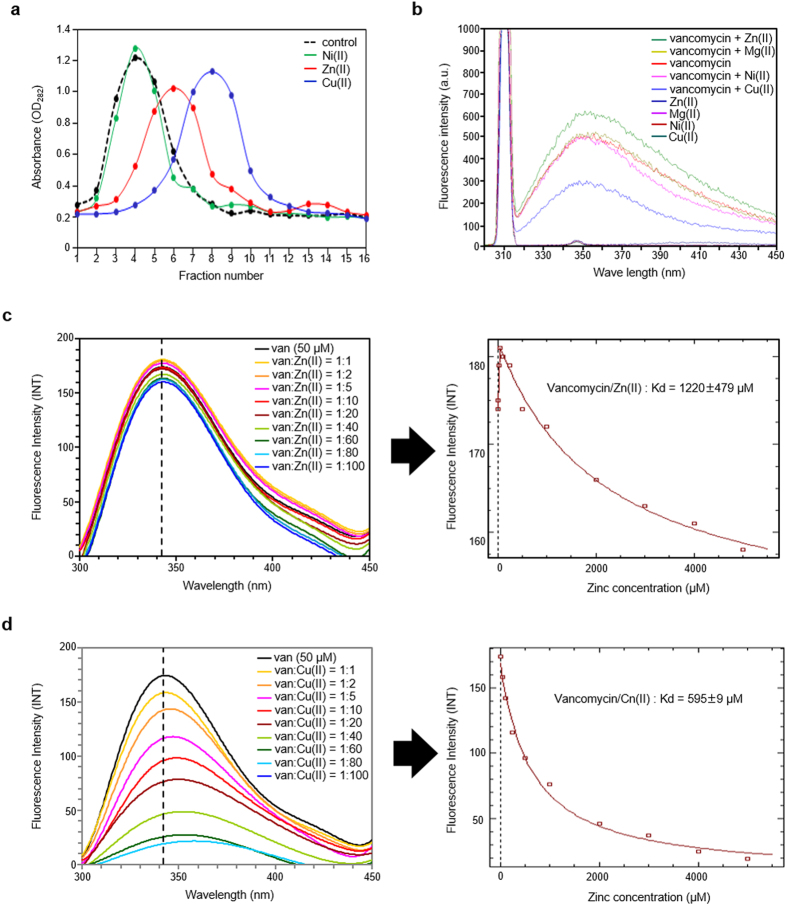
*In vitro* analysis of the interaction between vancomycin and Zn(II). (**a**) Affinity chromatography demonstrating that vancomycin binds to Zn(II) and Cu(II) but not to Ni(II). Vancomycin was applied to an uncharged HiTrap column (control) and to columns charged with Ni(II), Zn(II) or Cu(II). Elution of vancomycin from the columns was quantified by the analysis of 0.5 ml fractions using UV absorbance at 282 nm. (**b**) Analysis of the interaction of vancomycin with metal ions using fluorometry (λexcitation 280 nm, Slit: 5 nm, Tris-Cl pH 7.3). Cu(II) and Zn(II) alter the fluorescence emission of vancomycin but Ni(II) does not. (**c**) Analysis of the changes in fluorescence of vancomycin (50 μM) in the presence of increasing molar equivalent ratios of Zn(II) using optimized fluorescence conditions (λexcitation 280 nm, Slit: 5 nm, Tris-Cl pH 7.3). Fluorescence is most enhanced with a 1:1 ratio then progressively quenched with higher Zn(II) concentrations. Fluorescence titration data were analysed using DynaFit 4 to determine the equilibrium dissociation constant (Kd) for the vancomycin-Zn(II) binding (right panel). (**d**) Analysis of the changes in fluorescence of vancomycin (50 μM) in the presence of increasing molar equivalent ratios of Cu(II) using the same conditions as in (**c**). The wavelengths corresponding to the maximum fluorescence intensities in (**c**) and (**d**) are indicated by dotted lines.

To detect a possible physical interaction between vancomycin and Zn(II) we performed affinity column chromatography analysing the retention of vancomycin by a column charged with Zn(II) (Fig. 3a). Elution of vancomycin from the column was monitored both by UV spectrophotometry at 282 nm and by using a bioassay against a Δ*vanRS* vancomycin sensitive mutant strain (Supplementary Fig. 1). Retention by an uncharged column and a column charged with Cu(II) was also analysed as negative and positive binding controls, respectively. As expected, the column loaded with Cu(II) delayed the elution of vancomycin compared to the negative control. The zinc column also caused a marked delay but this was not as pronounced as the effect seen with Cu(II). This indicates that vancomycin can interact with Zn(II), but the binding affinity is weaker than for Cu(II) under these conditions. A column charged with Ni(II) produced the same behaviour as the negative control indicating that vancomycin does not generically interact with all divalent metal cations and providing evidence that, like the interaction with Cu(II)^14^,^17^, the binding between vancomycin and Zn(II) is specific.

Data supporting the interaction between vancomycin and Zn(II) was also obtained by analysing the inhibition of zinc-dependent calf intestinal phosphatase enzyme activity by vancomycin (Supplementary Fig. 2), and was conclusively demonstrated using fluorometry. Vancomycin is naturally fluorescent and exhibits an emission peak at ~350 nm when excited between 280–310 nm. Metal ion solutions however do not fluoresce in this range but physical interaction of metal ions with vancomycin can be observed through binding-induced changes in the emission spectrum of vancomycin produced by molecular conformational changes. Cu(II) has an established 1:1 binding ratio with vancomycin^14^ and causes a marked change in its fluorescence emission spectra between 300–450 nm. No significant change was observed in the presence of Mg(II) or Ni(II), but Zn(II) induced a strong effect (Supplementary Fig. 3). This indicates that vancomycin can therefore bind to both Cu(II) and Zn(II), but not to Mg(II) or Ni(II).

### Determination of the binding affinity between vancomycin and Zn(II) using a novel fluorometric assay

To determine the equilibrium dissociation constant (Kd) between vancomycin and Zn(II) we optimized the fluorometric assay used above, analysing changes in fluorescence when using 50 μM vancomycin in 100 mM Tris-Cl buffer at pH 7.3 with an excitation wavelength of 280 nm. These more carefully controlled conditions avoided the effect of pH variations on the fluorescence spectrum of vancomycin and reproduced the results observed in Supplementary Figure 3 (Fig. 3b). It also indicates that vancomycin fluorescence is enhanced at low Zn(II) concentrations but quenched at higher concentrations (Supplementary Fig. 4). Vancomycin fluorescence emission spectra were determined in the presence of a series of increasing Zn(II) ligand concentrations and the fluorescence data fitted to a model using DynaFit 4^18^ producing a Kd value of 1220 ± 479 μM (Fig. 3c). Similar quantification of the previously characterized interactions of vancomycin with D-Ala-D-Ala, D-Ala-D-Lac or Cu(II) yielded Kd values of 1.19 ± 0.59 μM, 1776 ± 589 μM and 595 ± 92 μM respectively (Supplementary Fig. 5 and Fig. 3d). The former two values are in good agreement with literature values while this is the first report of the Kd for Cu(II) binding to vancomycin. Isothermal titration calorimetry (ITC) determined the Kd for vancomycin and Zn(II) to be 1007 μM, but 1.63 μM between vancomycin and D-Ala-D-Ala and 3745 μM between vancomycin and D-Ala-D-Lac (Supplementary Fig. 6). The ITC and fluorometry data are therefore in good agreement and the order of the binding affinities between vancomycin and the tested ligands is D-Ala-D-Ala > Cu(II) > Zn(II) > D-Ala-D-Lac.

### Characterization of Zn(II) binding to vancomycin using solution NMR

**Figure 4:**
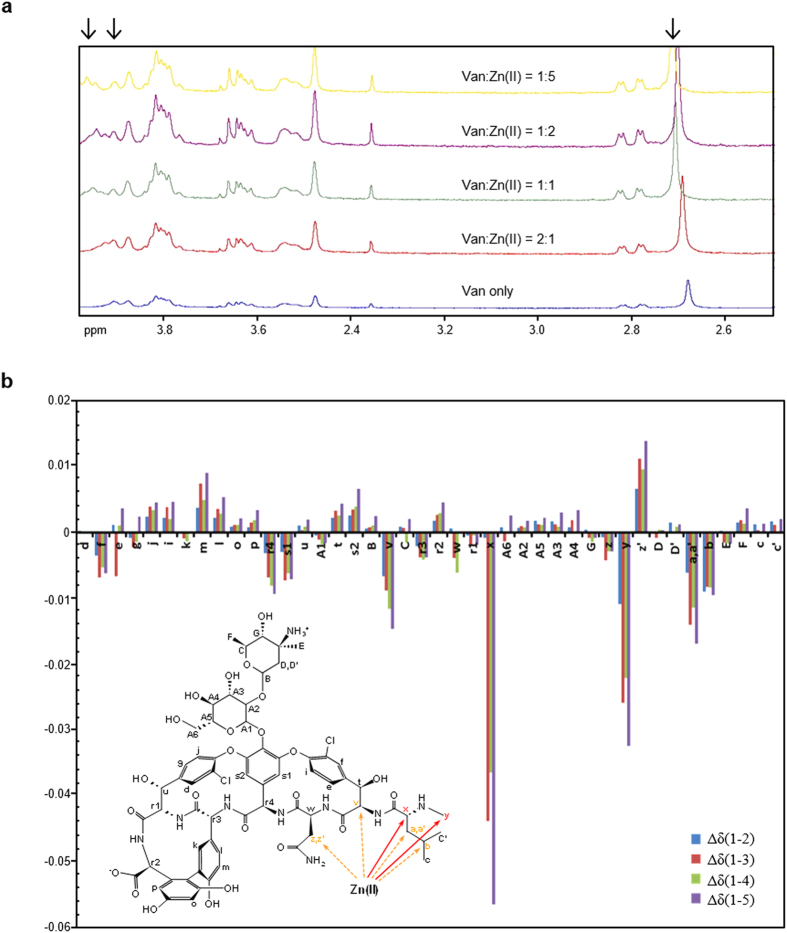
^1^H NMR analysis of Zn(II) binding to the vancomycin structure in deuterium oxide (D_2_O) at pD7.1. (**a**) The chemical shift (ppm) of certain proton resonances in the ^1^H NMR spectrum of vancomycin are affected by increasing concentrations of Zn(II) ions. The arrows indicate the resonances changing in the 2.5 to 4 ppm spectral region as the concentration of Zn(II) present is increased from 0 mM (bottom spectrum, 1 mM vancomycin) to 5 mM (top spectrum, Van: Zn(II) = 1:5). (**b**) Complete shift analysis summarizing the difference in proton resonance shifts between each zinc condition and the control. The differences between the control (0 mM Zn(II)) and 0.5 mM (Van:Zn(II) = 2:1), 1 mM (Van:Zn(II) = 1:1), 2 mM (Van:Zn(II) = 1:2) and 5 mM (Van:Zn(II) = 1:5) zinc chloride are named Δδ(1–2), Δδ(1–3), Δδ(1–4) and Δδ(1–5) respectively. The largest changes are mapped onto the vancomycin structure defining the Zn(II) binding site.

To define the binding of Zn(II) to vancomycin at the molecular level we determined the effect of incremental levels of Zn(II) on the ^1^H NMR structure of vancomycin in deuterium oxide (D_2_O; pD 7.1) (Fig. 4). We applied the proton naming convention and method of spectral calibration used by Swiatek *et al.*^17^. Figure 4a highlights vancomycin proton resonances in the 2.6–4 ppm region which shift subtly with the increasing presence of zinc, while Fig. 4b summarizes all the differences in shifts (Δδ = δ_bound_ − δ_free_) observed for each resonance between the control spectrum (no zinc) and the spectra from vancomycin in the presence of 0.5 mM, 1 mM, 2 mM and 5 mM Zn(II). Previous NMR studies characterizing Zn(II)-peptide binding indicate that a resonance shift (Δδ) greater than 0.03 ppm is consistent with a significant interaction^19^. Using this criteria, our vancomycin-Zn(II) NMR analysis indicates that signals for CH(x) and CH_3_(y) were significantly shifted (≥0.03 ppm). Four other resonances, corresponding to protons CH_2_(a,a’), CH(b), CH(v) and CH(z’), exhibited clear shifts but with Δδ values between 0.01 and 0.02 ppm. These protons are all located in the same region of vancomycin and thus indicate the likely Zn(II) binding site. This putative Zn(II) binding site is similar to the site identified for Cu(II) by Kucharczyk *et al.*^14^.

## Discussion

Four different lines of evidence indicate that vancomycin can bind to Zn(II) *in vitro*: i) it is retained on an affinity column charged with Zn(II) (Fig. 3a); ii) it can inhibit the activity of an enzyme for which Zn(II) is an essential co-factor (Supplementary Fig. 2); iii) its fluorescence in solution is altered by Zn(II) (Fig. 3b,c, Supplementary Figs 3 and 4); and iv) a subset of its protons resonate at different chemical shifts in the presence of Zn(II) (Fig. 4). *In vivo*, vancomycin exposure induces a zinc starvation response in bacteria as diverse as *Streptomyces* species, *E. coli* and *B. subtilis* (Fig. 2). These data are entirely consistent with the external presence of vancomycin sequestering Zn(II) from cellular use and activating an adaptive response aimed at restoring intracellular Zn(II) to normal levels. Bacterial cells are impermeable to vancomycin, which exerts its antibiotic activity in the cell wall matrix by non-covalently binding to the D-Ala-D-Ala termini of lipid II units, and the diversity of the genetic backgrounds and cellular properties of the bacterial species affected in this study also makes alternative explanations unlikely. Vancomycin is not active against Gram-negative bacteria due to an inability to penetrate their outer membranes, and the induction of zinc starvation by vancomycin in *E. coli* therefore occurs independently of antibiotic activity.

The Kd of vancomycin for Zn(II) was calculated as 1220 μM using a new method based on solution fluorometry. This value is approximately double the 595 μM value calculated in this study for the interaction with Cu(II) using the same methodology, indicating a significantly weaker interaction that is consistent with the relative retention of vancomycin by Zn(II) or Cu(II) charged affinity columns (Fig. 3a). It is also consistent with the relative stability of complexes formed by Cu(II) and Zn(II) according to the Irving-Williams series^20^. The observation that vancomycin fluorescence is initially enhanced at low Zn(II) concentrations but quenched at higher concentrations (Fig. 3c, Supplementary Fig. 4) could in principle suggest a two order binding process, and possibly two binding sites for Zn(II), but this was not supported by either the ITC or NMR data collected and the binding constants calculated are therefore for a first order process. The binding site of Zn(II) to vancomycin identified using proton NMR spectroscopy is similar to that previously reported for Cu(II)^14^,^17^. Cu(II) forms a square planer coordination complex with three nitrogen atoms and one oxygen atom that span amino acids 1–3 of vancomycin. The observed resonance shifts for vancomycin-Zn(II) are consistent with a similar binding site, although Zn(II) typically prefers tetrahedral coordination geometry^21^ and the exact nature of the binding is therefore likely to be different. The differing effects of Cu(II) and Zn(II) on the vancomycin fluorescence emission spectrum also suggests dissimilarities in the nature of binding. Cu(II) produced a red shift in the emission spectrum (Fig. 3d), while Zn(II) binding altered the fluorescence intensity without causing any noticeable shift (Fig. 3b,c, Supplementary Fig. 4). Binding of D-Ala-D-Ala or D-Ala-D-Lac both produced a blue shift (Supplementary Fig. 5).

The ability of external vancomycin to induce intracellular zinc starvation is remarkable given that *in vitro* it binds Zn(II) with millimolar affinity, yet the *S. coelicolor* Zur protein has a graded binding response to sub-femtomolar intracellular concentrations of Zn(II)^22^. In contrast, EDTA is a Zn(II) chelator with a sub-femtomolar Kd^23^, but EDTA treatment of bacterial cultures induced zinc starvation responses of a similar order of magnitude to vancomycin (Fig. 2). This disparity between *in vitro* and *in vivo* results could be due to competition for EDTA binding with other metal ions (particularly Ca(II) which has a slow binding off-rate) in the *in vivo* experiment, but could also reflect potential differences in the spatial distribution of the chelators in the cultures. Vancomycin is likely to be specifically highly concentrated in the immediate cell environment due to its interaction with D-Ala-D-Ala residues in the peptidoglycan (Kd between vancomycin and D-Ala-D-Ala ~1.6 μM) and this is likely to potentiate its effect on the uptake of Zn(II), bringing the localized concentration of vancomycin into a range where it can exert a significant influence on zinc bioavailability despite its high Kd. Bacterial cell walls can also contain a significant proportion of the total cellular zinc, e.g. 40–60% in some *Streptomyces* species^24^, potentially exacerbating this effect. We also cannot exclude the possibility that the binding affinity of vancomycin for Zn(II) can be strengthened by the involvement of an unknown chemical present in the cell matrix that is absent from the *in vitro* binding studies.

## Methods

### Bacterial culture and RNA preparation

*S. coelicolor* M600, *S. griseus* IFO 13350, *S. roseosporus* NRRL15998, *E. coli* BL21(DE3) and *B. subtilis* JH642 were all grown to mid-exponential phase in a minimal liquid medium supplemented with ~3 μM zinc sulphate and sampled to obtain untreated control cells. The cultures were then immediately divided into two aliquots and treated with either 2 mM of vancomycin or EDTA for 30 min before sampling again.

RNA preparation from *Streptomyces* strains were performed according to the method described previously^8^,^9^. Spores of each *Streptomyces* strain were germinated by heat shock treatment in TES buffer and incubated in double-strength germination medium at 37 °C for 3 h. 0.5 ml of germinated spores were then inoculated in 50 ml NMMP liquid medium (containing approximately 3 μM zinc sulphate)^25^ and grown to mid-exponential phase (OD_450_ of 0.3–0.6) at 30 °C in an orbital shaker set at 250 rpm. Immediately after the first 10 ml of sample was taken as the non-induced control, the remaining culture broth was divided into two aliquots and treated with either 2 mM of vancomycin or EDTA for 30 min with shaking before sampling again. Sampling was performed by centrifugation at 4,000 g for 1 min, resuspending the cell pellet in 10 ml of sterile 10.3% sucrose solution to wash. After washing the cells were pelleted as before and flash frozen in liquid nitrogen. Cell pellets were stored at −80 °C until use. For RNA preparation, cell pellets were resuspended in 1 ml of ice-cold Kirby mix^25^ and sonicated twice for 4–5 s each, then extracted with 0.8 ml of phenol-chloroform (pH 8.0) by vortexing. Samples were re-extracted with phenol-chloroform (pH 8.0) and nucleic acids were precipitated at −20 °C using 0.3 M sodium acetate (pH 6.0) and an equal volume of isopropanol. After centrifugation, the nucleic acids pellet was washed with 70% ethanol, dissolved in DNase I buffer, then treated with 5 units of DNase I at 37 °C for 30 min. Samples were extracted with phenol-chloroform (pH 8.0) and precipitated again as described above. After centrifugation, RNA pellets were dissolved in RNase-free distilled water and stored at −80 °C.

*E. coli* cells were grown in M9 minimal media (composition per liter, 2 mM MgSO_4_, 0.1 mM CaCl_2_, 0.4% glucose, 12.8 g Na_2_HPO_4_·7H_2_O, 3 g KH_2_PO_4_, 0.5 g NaCl, 1 g NH_4_Cl)^26^ supplemented with 3 μM zinc sulphate. *B. subtilis* cells were grown in Spizizen’s minimal medium (SMM) (composition per liter, 2 g (NH_4_)_2_SO_4_, 14 g K_2_HPO_4_, 6 g KH_2_PO_4_, 1 g Na_3_-citrate·2H_2_O, 0.2 g MgSO_4_·7H_2_O)^27^ supplemented with 3 μM zinc sulphate, 0.5% glucose and two required amino acids, 20 mg l^−1^ L-tryptophan and 18 mg l^−1^ L-phenylalanine. Both bacteria were grown to OD_600_ of 0.6–0.8 at 37 °C in orbital shaker with 250 rpm and the 10 ml of first samples were taken as an untreated control. The rest of culture broth was then divided into two aliquots and treated with either 2 mM vancomycin or EDTA for 30 min with shaking at 37 °C before sampling. All samples was centrifuged for 10 min at 4,000 g. For RNA preparation, RNeasy Protect Mini Kit (Qiagen) was used according to the manufacturer’s instructions.

### qRT-PCR

qRT-PCR experiments were carried out as described previously^9^. All primers used for pRT-PCR analysis in this study were designed using Primer3 ( http://primer3.ut.ee/) and are listed in Supplementary Table 1. For cDNA synthesis, the DNaseI-treated RNA was used as template in a 20 μl reaction volume employing Superscript III First Strand Synthesis Supermix (Invitrogen). PCR cycling was performed at 25 °C for 10 min, 42 °C for 120 min, 50 °C for 30 min, 55 °C for 30 min and then 85 °C for 5 min. Each PCR product was then treated with RNaseH and diluted in DNase-free distilled water. 2.5–5 μl aliquots of each cDNA sample were subjected to qRT-PCR analysis in total 25 μl reaction volumes with SYBR GreenER qPCR Supermix (Invitrogen) according to the manufacturer’s instructions. Each 25 μl reaction solution contained 12.5 μl of SYBR GreenER qPCR Supermix, 200 nm Rox, 250 nm of forward and reverse primers and 3 μl of 40% DMSO. qRT-PCR cycling was performed in a 7300 Real Time PCR System (Applied Biosystems) at 50 °C for 2 min, 95 °C for 10 min, followed by 30 cycles of 95 °C for 15 s and 57 °C for 1 min. The results were analysed using 7300 system SDS Software (version 1.4, Applied Biosystems). qRT-PCR determinations were performed in triplicate on each RNA sample and average abundances used. All qRT-PCR results are presented in the log scale. Values determined for *S. coelicolor* genes were normalized to the endogenous control gene SCO4742^5^, while qRT-PCR results in all other bacterial strains were normalized to their own gene encoding 16S rRNA except *S. roseosporus* results which were normalized to the *rpsL* gene encoding a 12S ribosomal protein^28^.

### Metal affinity column chromatography

HiTrap Chelating HP (GE healthcare) columns were prepared by washing with 5 ml of deionized (DI) water at a flow rate of 1 ml/min. Washed columns were then loaded with 500 μl of 100 mM copper, nickel or zinc sulphate solutions as required, or left uncharged as a negative control. Columns were washed with 5 ml of DI water and then equilibrated with 10 ml of binding buffer (20 mM NaH_2_PO_4_/Na_2_HPO_4_ (pH 7.4), 200 mM NaCl, 10% Glycerol, 0.1% Triton-X-100). 150 μl of 10 mM vancomycin was applied to each column and elution performed using 5 ml of binding buffer followed by 5 ml of elution buffer (binding buffer supplemented with 50 mM imidazole). The first 1.5 ml of eluate was discarded before collecting sixteen 0.5 ml fractions. The relative abundance of vancomycin in each fraction was measured by UV spectrophotometry by diluting a 0.1 ml aliquot of each 0.5 ml fraction with 0.4 ml water and measuring the absorbance at 282 nm. A 20 μl aliquot of each diluted sample was also analysed in a bioassay by applying to freshly prepared MMCGT bioassay plates seeded with ~10^7^ spores of a vancomycin-sensitive *S. coelicolor* Δ*vanRS* strain (J3201)^29^, scoring the results after incubation at 30 °C for 2 days.

### Fluorometry assay

Fluorescence spectroscopy (PerkinElmer LS55, Waltham, MA, USA) was used to measure the fluorescence emission spectra of vancomycin hydrochloride (Sigma-Aldrich) solutions (0.5 ml). For the binding analysis presented in Supplementary Figure 3, 100 mg ml^−1^ vancomycin was used with the following parameters: 310 nm excitation wavelength; 2.5 nm slit width; 300–450 nm emission scan range; 50 nm min^−1^ scan speed. Zinc, copper, nickel or magnesium sulphate were added as required from 50 mM stock solutions in DI water. For the binding analysis presented in Fig. 3b,c and Supplementary Figure 4, 50 μM vancomycin and metal sulphate solutions were prepared in 100 mM Tris-Cl buffer (pH 7.3) and used along with the following optimized conditions: 280 nm excitation wavelength; 5 nm slit width; 300–450 nm emission scan range; 50 nm min^−1^ scan speed with best-fit trend-line smoothing. For quantifying the binding affinities, scans were measured in triplicate and the equilibrium dissociation constants (Kd) between vancomycin and Zn(II), D-Ala-D-Ala, D-Ala-D-Lac or Cu(II) were determined by analysis with DynaFit 4^18^.

### NMR

The ^1^H NMR experiments were performed using an AVANCE III 400MHz Bruker NMR spectrometer at a temperature of 328 K^17^. The solution pD was adjusted to 7.1 prior to scanning using sodium deuteroxide or deuterium chloride, and 150 scans recorded for each spectrum. Data were acquired for 1 mM vancomycin in the presence of 0, 0.5, 1, 2, and 5 mM of zinc chloride in deuterium oxide and processed with Bruker TopSpin 3.1. Proton resonances were assigned using the protocol of Swiatek *et al.*^17^, defining the shift for the invariant proton H_d_ as 7.74 ppm in all spectra. Complete shift analyses were performed by comparing the proton resonances of vancomycin with and without added zinc chloride.

## Additional Information

**How to cite this article**: Zarkan, A. *et al.* The frontline antibiotic vancomycin induces a zinc starvation response in bacteria by binding to Zn(II). *Sci. Rep.* **6**, 19602; doi: 10.1038/srep19602 (2016).

## Supplementary Material

Supplementary Information

## Figures and Tables

**Figure 1:**
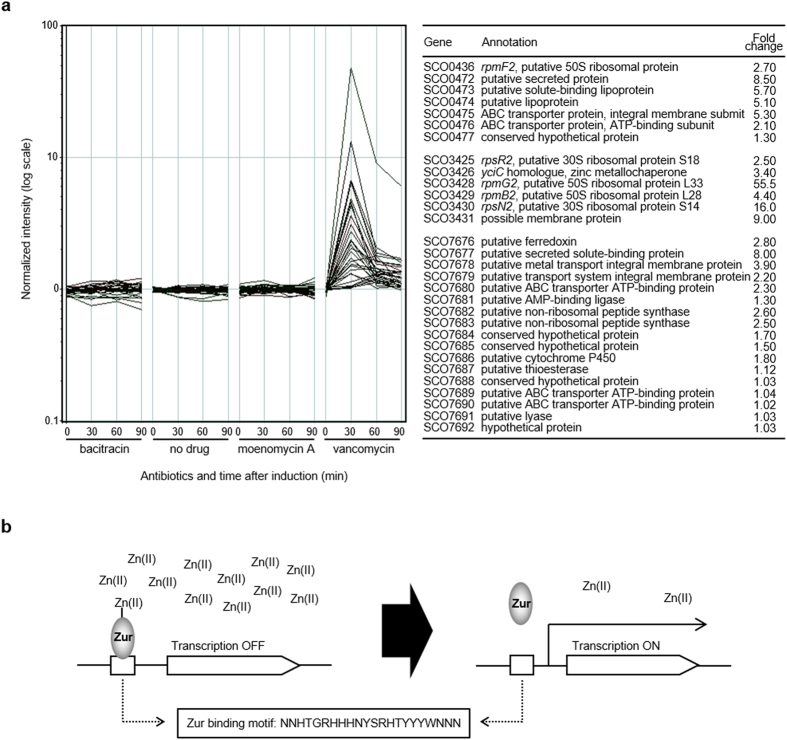
Evidence for the activation of Zur-dependent zinc homeostasis systems by vancomycin. (**a**) Zur-regulated genes specifically induced by 10 μg/ml vancomycin (6.9 μM) in the transcriptome of *S. coelicolor*^5^. Fold change corresponds to the change in expression of each gene 30 min after vancomycin treatment relative to time 0. (**b**) Regulation of gene expression by Zur in response to Zn(II). In high Zn(II) concentrations, zinc-bound Zur represses transcription of its target genes by binding to a Zur binding site in the promoter region. When Zn(II) is low, zinc-free Zur is released from the promoter allowing transcription.
