# Supplementary material for: The frontline antibiotic vancomycin induces a zinc starvation response in bacteria by binding to Zn(II)
Source: Sci Rep. 2016 Jan 22;6:19602. doi: 10.1038/srep19602 (PMC4726154; doi:10.1038/srep19602)
Supplement: Supplementary Information [file srep19602-s1.pdf]

SUPPLEMENTARY INFORMATION

The frontline antibiotic vancomycin induces a zinc starvation response in bacteria by binding to Zn(II)

Ashraf Zarkan[1], Heather-Rose Macklyne[1], Andrew W. Truman[2], Andrew R. Hesketh[1,3], and Hee-Jeon Hong[1,*]

[1]Department of Biochemistry, University of Cambridge, Cambridge, CB2 1QW, UK

[2]Department of Molecular Microbiology, John Innes Centre, Norwich, NR4 7UH, UK

[3]Cambridge Systems Biology Centre, University of Cambridge, Cambridge, CB2 1QW, UK

*Correspondence: hh309@cam.ac.uk

Supplementary Methods

Calf Intestinal Alkaline Phosphatase (CIAP) activity assay

CIAP is a metalloprotein which requires Zn(II) as a cofactor for its activity[1, 2, 3]. CIAP dephosphorylates colourless p-nitrophenylphosphate (pNPP) to release the yellow coloured reaction product p-nitrophenyl (pNP). The rate of colour evolution is proportional to the enzyme activity and can be quantified at 415 nm. To measure the effect of vancomycin and EDTA on CIAP enzyme activity, aliquots (50 µl) of a concentration series of vancomycin or EDTA (0, 1, 2, 5, 10, 15, 20, 50, 100 mM) were added to aliquots (50 µl) of CIAP enzyme (0.1 units in the supplied reaction buffer; New England Bioloabs) in a 96 well plate then mixed with pNPP substrate (Sigma-Aldrich; 100 µl of a 0.5 mg ml-1 solution in 50 mM Tris-Cl, pH 8). Two negative controls containing 100 mM vancomycin or EDTA with pNPP substrate but no CIAP were also used. pNP formation was quantified at 415 nm at regular intervals over 90 min using a Bio-Rad 680 Microplate Reader. When present in excess, Zn(II) is known to inhibit CIAP enzyme activity[4, 5]. This phenomenon can also be exploited to analyze the chelation of Zn(II) by vancomycin by assaying for a restoration of enzyme activity after pretreatment of CIAP with excess Zn(II). Aliquots (50 µl) of CIAP (0.1 units in the supplied reaction buffer; New England Bioloabs) were prepared in 1 mM zinc sulphate to inactivate the enzyme and dispensed into a 96 well plate. These were mixed with aliquots (50 µl) of a concentration series of vancomycin (0, 0.5, 1, 2, 4, 6, 8, 10 mM) or EDTA (0, 0.5, 1, 2, 4, 6, 8, 10 mM) and the reaction started by addition of the pNPP substrate (Sigma-Aldrich; 100 µl of a 0.5 mg ml-1 solution in 50 mM Tris-Cl, pH 8). Two negative controls containing 100 mM vancomycin or EDTA with pNPP substrate but no CIAP were also added. pNP formation was again quantified at 415 nm at regular intervals over 90 min using a Bio-Rad 680 Microplate Reader.

Isothermal Titration Calorimetry (ITC)

Isothermal titration calorimetry experiments were carried out using a MicroCal ITC200 Microcalorimeter. All solutions were made using 50 mM Tris-Cl pH 7.2 and experiments were conducted at 298 K. Binding affinity with D-Ala-D-Ala was assessed by titrating N,N'-Ac2-Lys-D-Ala-D-Ala (3.33 mM in 38 µl buffer) into vancomycin (0.3 mM in 200 µl buffer) as 59 x 0.5 µl injections with 120 s spacing. D-Ala-D-Lac affinity was assessed by titrating N,N'-Ac2-Lys-D-Ala-D-Lac (6.66 mM in 38 µl buffer) into vancomycin (0.3 mM in 200 µl buffer) as 26 x 1.5 µl injections with 120 s spacing. Zn(II) affinity was assessed by titrating zinc chloride (5 mM in 40 µl) into to vancomycin (0.3 mM in 200 µl buffer) as 19 x 2 µl injections. Control titrations were carried out for all ligands by titrating into buffer only. Origin 7 (OriginLab) was used for all data analysis and curve fitting. N was fixed as 1 to assist with curve fitting for the vancomycin:Zn(II) interaction.

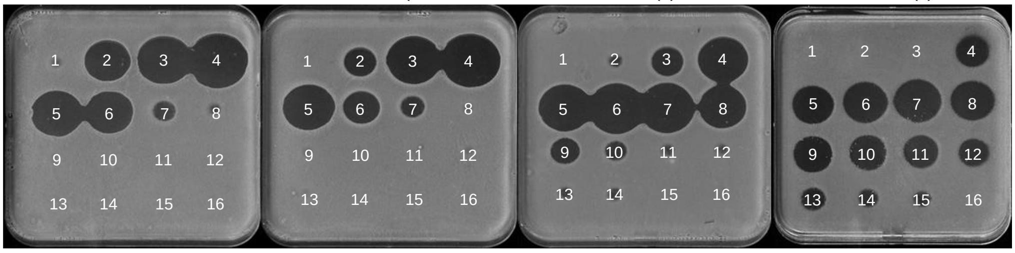

Supplementary Figure 1: Bioassay analysis of the fractions eluted from the affinity column chromatography presented in Fig. 3a.

Vancomycin was applied to an uncharged HiTrap column (control) and to columns charged with Ni(II), Zn(II) or Cu(II). Elution of vancomycin from the columns was quantified in a bioassay against a vancomycin sensitive *S. coelicolor* Δ*vanRS* mutant strain. A halo of non-growth around the spotted fractions indicates vancomycin, and halo size is proportional to the amount present.

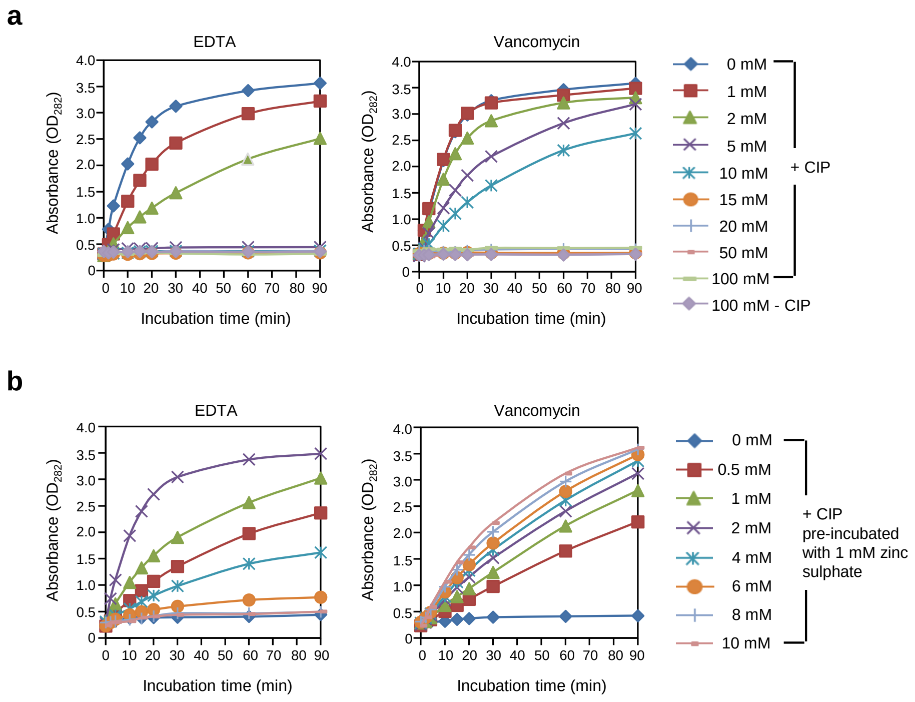

Supplementary Figure 2: A CIAP enzyme activity assay indicates the chelation of Zn(II) by vancomycin.

(a) Concentrations of EDTA in excess of 5 mM completely inhibit CIAP activity presumably through sequestration of the zinc cofactor. Vancomycin shows a similar inhibitory activity against CIAP although the effect is weaker and >15 mM is required for complete inhibition. (b) Both EDTA and vancomycin are capable of restoring CIAP enzyme activity suggesting chelation of Zn(II). Addition of 2 mM EDTA completely restores activity but increasing the concentration above that limit starts to produce the inhibitory effect observed in the first assay above. Increasing concentrations of vancomycin from 0-10 mM produces a stepwise increase in CIAP activity although maximal activity is never restored. This is again consistent with vancomycin being capable of chelating Zn(II) but with much lower affinity than EDTA.

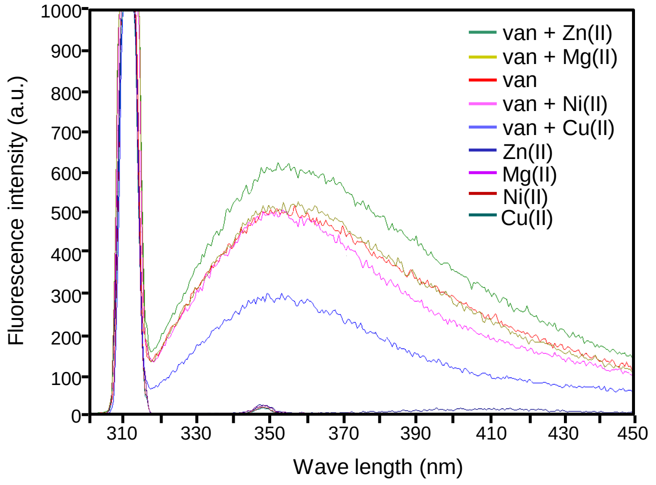

Supplementary Figure 3: Analysis of the interaction of vancomcyin with metal ions using fluorometry (λexcitation 310 nm, Slit: 2.5 nm, dH2O).

Cu(II) and Zn(II) markedly alter the fluorescence emission of vancomycin but Mg(II) and Ni(II) do not. No emission was observed when each metal ion was tested alone.

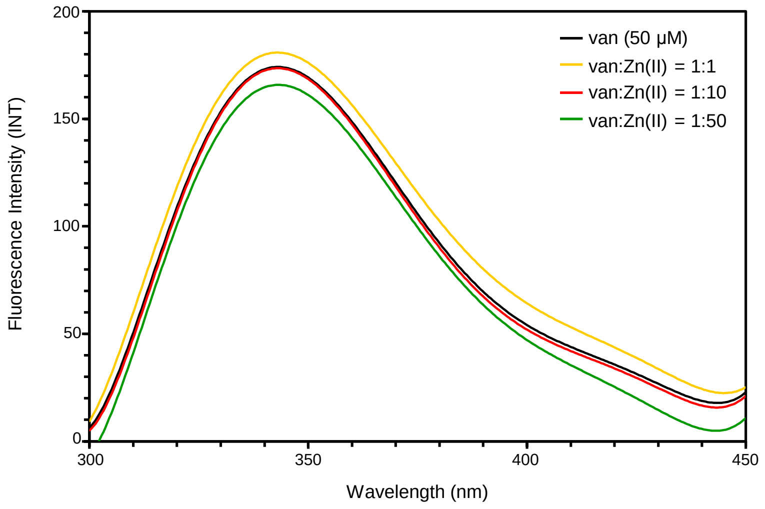

Supplementary Figure 4: The fluorescence of vancomycin is enhanced by the addition of low Zn(II) concentrations (Zn(II) up to a 1:1 molar ratio) but quenched at higher Zn(II) concentrations.

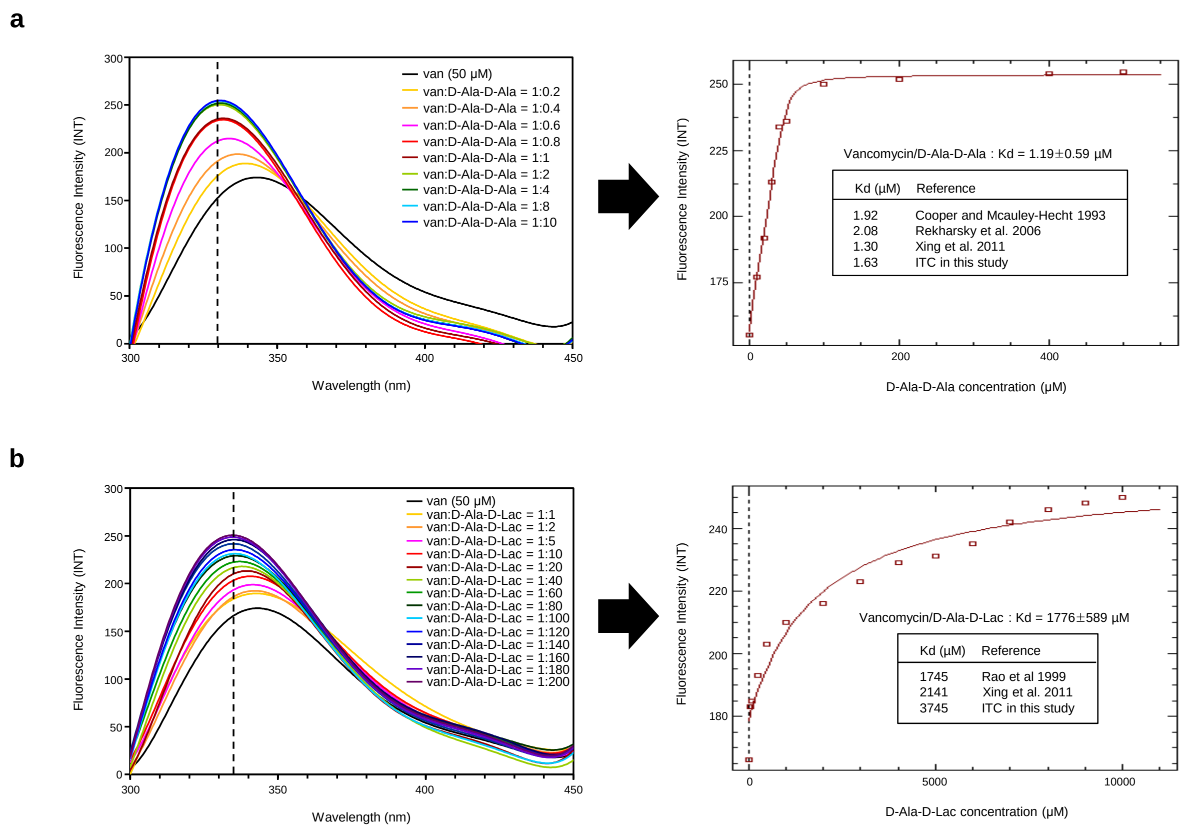

Supplementary Figure 5: Fluorometry emission spectra (λexcitation 280 nm, Slit: 5 nm, Tris-Cl pH 7.3) of vancomycin alone and with added concentrations of Ac-Lys(Ac)-D-Ala-D-Ala-OH (D-Ala-D-Ala) or Ac-Lys(Ac)-D-Ala-D-Lactic Acid (D-Ala-D-Lac).

Using the fluorometry assay to calculate the equilibrium dissociation constants for the interaction of vancomycin with D-Ala-D-Ala (a) and D-Ala-D-Lac (b). In each case the fluoresence emission spectrum in the presence of a range of ligand concentrations were recorded and the fluoresence intensity at the peak emission wavelength plotted against the concentration of ligand used (panels to the right) and the data analyzed using DynaFit 4.The Kds in (a) and (b) are highly similar to previously published values and to values determined here via ITC (see inset panels). The wavelengths corresponding to the maximum fluorescence intensities in (a) and (b) are indicated by dotted lines.

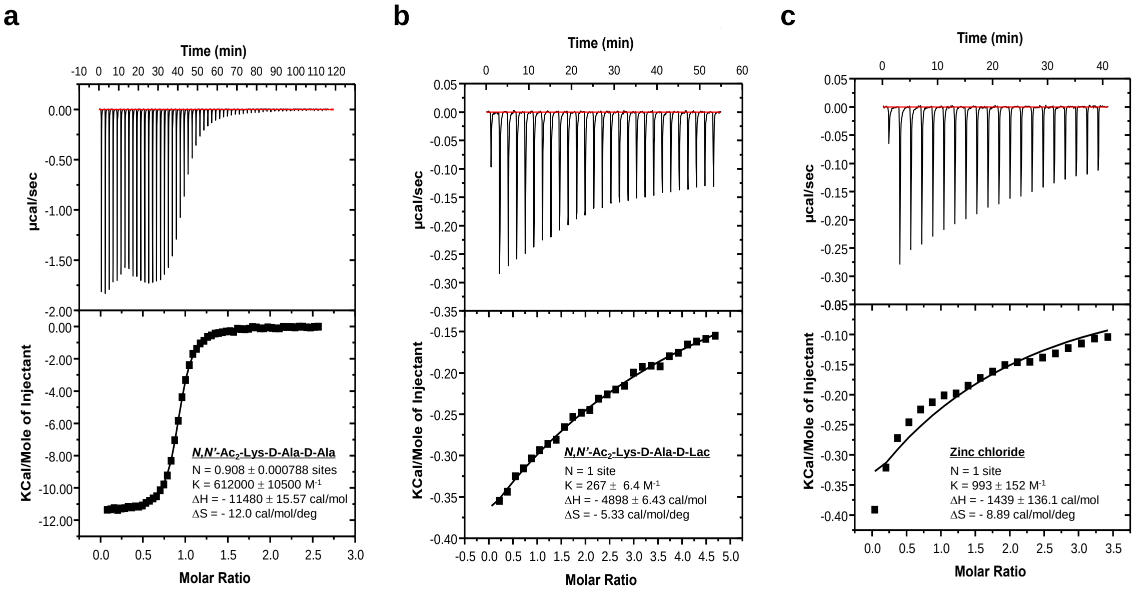

***N,N'*-Ac$_2$-Lys-D-Ala-D-Ala : Vancomycin**

	K/M^{-1}	ΔH (kJ/mol)	ΔS (J/mol/K)	ΔG (kJ/mol)
This study	612000	-48.0	-50.2	-33.1
2011 FCS	769230	-	-	-33.9
2006 ITC	480000	-40.2	-25.8	-32.5
1993 ITC	520000	-53.3	-69.0	-33.7

***N,N'*-Ac$_2$-Lys-D-Ala-D-Lac : Vancomycin**

	K/M^{-1}	ΔH (kJ/mol)	ΔS (J/mol/K)	ΔG (kJ/mol)
This study	267	-20.5	-22.2	-13.9
2011 FCS	467	-	-	-15.1
1999 ITC	573	-15.5	-1.40	-15.1

Zn(II) : Vancomycin

	K/M^{-1}	ΔH (kJ/mol)	ΔS (J/mol/K)	ΔG (kJ/mol)
This study	993	-6.02	-37.2	-5.07

Supplementary Figure 6: Isothermal titration calorimetry analysis of vancomycin with *N*,*N*'-Ac$_2$-Lys-D-Ala-D-Ala (a), *N*,*N*'-Ac$_2$-Lys-D-Ala-D-Lac (b) and zinc chloride (c). 1993 ITC study refers to Cooper and McAuley-Hecht[6]. 1999 ITC study refers to Rao *et al*.[7]. 2006 ITC study refers to Rekharsky *et al*.[8]. 2011 FCS (fluorescence correlation spectroscopy) study refers to Xing *et al*.[9].

Supplementary Table 1: Primers for qRT-PCR used in this study.

strain	gene	primer name	sequence (5'-3')
S. ceolicolor M600	SCO4742*	q4742 For<br>q4742 Rev	GGAGGTACCGGTGCTGATC<br>GGGTCATCAGCGTCGGTG
	SCO0475	q0475 For<br>q0475 Rev	GTGACGTTTGTGCAAAGAGC<br>AGATGGCGGATACCAGGAT
	SCO0476	q0476 For<br>q0476 Rev	GTACGTCTGACCGAGCTGTG<br>CAGGTGAGTTGCCTCAGGA
	SCO2505 (= *znuA*)	q2505 For<br>q2505 Rev	GCCACCAAGGTCTTCTTCAC<br>CTGCTGGAGTTCCTTGATCC
	SCO7676	q7676 For<br>q7676 Rev	TTCGACCAGGACGAGGAG<br>GAAGAGTGAGCGCTCCAGA
	SCO7677	q7677 For<br>q7677 Rev	GACCCTCGACTTCTTCAACG<br>ATGTCGATTCCCTTGAGCAC
	SCO7681	q7681 For<br>q7681 Rev	ACACGTTGCGTGGCTACTAC<br>GGTTGATCTGGTCCTTGAGC
	SCO7682	q7682 For<br>q7682 Rev	CCGTACTGCACCTCTCCTTC<br>ACGTAGTCGCGGAAAGTGAT
S. griseus IFO13350	SGR16S rRNA1*	qrRNA1 For<br>qrRNA1 Rev	AGTAATGGCCCAGAGATCCG<br>AGCTAGAGTGTGGTAGGGGA
	SGR5019 (= SCO2505)	q5019 For<br>q5019 Rev	CCCTTGAGGTACAGCACGTA<br>AGTTCCTGGCCGAGAAGATC
	SGR0545 (= SCO3429)	q0545 For<br>q0545 Rev	CTGTCGACGGTCCTGATGG<br>CAACAACATCTCCCACTCGC
	SGR0546 (= SCO3428)	q0546 For<br>q0546 Rev	AAGTACGCCCGGTCATCAAG<br>TCGTTCCGCCGGTTCTTG
S. roseosporus NRRL15998	*rpsL* (= ribosomal protein 12S)*	qrpsL For<br>qrpsL Rev	GTCCGCAGTATTCGACACAC<br>GCGTCTTGTTCTTCTCGACC
	SSGG01683 (= SCO2505)	q01683 For<br>q01683 Rev	GGAAAGTCGCTGGAGAAGG<br>TGGTGATGAAGGTCTTGGTG
	SSGG00253 (= SCO0476)	q00253 For<br>q00253 Rev	CAACTCAGCGCCGCAATAC<br>ATGTGGCGGTGATCACTCC
	SSGG06641 (= SCO3428)	q06641 For<br>q06641 Rev	TCGTTCCGCCGGTTCTTAC<br>AAGTACGCCCGGTCATCAAG
E. coli BL21(DE3)	*rrsA* (= 16s rRNA)*	qrrsA For<br>qrrsA Rev	CGATCCCTAGCTGGTCTGAG<br>TTCTTCATACACGCGGCATG
	znuA	qznuA For<br>qznuA Rev	TGGCTTTCCCCAGAGATAGC<br>GCGTTTCGGTTGAGGCTAAT
	znuB	qznuB For<br>qznuB Rev	TGGTCATCGTGGTGGCTATT<br>CACCATCAACAAACGCCAGA
	znuC	qznuC For<br>qznuC Rev	GCGTGTACTATTAGCGCGAG<br>CCTGGCCATTTACATCCACG
B. subtilis JH642	*rrnA1* (=16s rRNA)*	qrRNA For<br>qrRNA Rev	AGGTGGGGATGACGTCAAAT<br>ACTGCGATCCGAACTGAGAA
	znuA (= *ycdH*)	qycdH For<br>qycdH Rev	AGTCATGCAATGGACCCTCA<br>TTGTTATCCGGGTCCTGCTT
	znuB (= *yciA*)	qyciA For<br>qyciA Rev	GGTATTGAAGCGGCCTGATG<br>TCTCAAAGGCTGACACCCAT
	znuC (= *yciC*)	qyciC For<br>qyciC Rev	AGAAATCGCCGACCTCTTGA<br>GGAACGAATGAACCTGGCTC

* Indicates the gene used as the normalization reference in each species.

Supplementary References

1. Bortolato, M., Besson, F. & Roux, B. Role of metal ions on the secondary and quaternary structure of alkaline phosphatase from bovine intestinal mucosa. *Proteins* **37**, 310-318 (1999).
2. Stec. B., Holtz, K. M. & Kantrowitz, E. R. A revised mechanism for the alkaline phosphatase reaction involving three metal ions. *J. Mol. Biol*. **299**, 1303-1311 (2000).
3. Zhang, L., Buchet, R. & Azzar, G. Distinct structure and activity recoveries reveal differences in metal binding between mammalian and *Escherichia coli* alkaline phosphatases. *J. Biochem*. **392**, 407-415 (2005).
4. Rej, R. & Bretaudiere, J. P. Effects of metal ions on the measurement of alkaline phosphatase activity. *Clin. Chem*. **26**, 423-428 (1980).
5. Yan, S. *et al*. Effect of extraneous zinc on calf intestinal alkaline phosphatase. *J. Protein Chem*. **22**, 371-375 (2003).
6. Cooper, A. & McAuley-Hecht, K. E. Microcalorimetry and the Molecular Recognition of Peptides and Proteins. *Phil. Trans. R. Soc. Lond. A* **345**, 23-35 (1993).
7. Rao, J. *et al*. Binding of a dimeric derivative of vancomycin to L-Lys-D-Ala-D-lactate in solution and at a surface. *Chem. Biol*. **6**, 353-359 (1999).
8. Rekharsky, M. *et al*. Thermodynamics of interactions of vancomycin and synthetic surrogates of bacterial cell wall. *J. Am. Chem. Soc*. **128**, 7736-7737 (2006).
9. Xing, B. *et al*. Molecular interactions between glycopeptide vancomycin and bacterial cell wall peptide analogues. *Chem. Eur. J*. **17**, 14170-14177 (2011).
